# BioHarness: substrate-aware evidence assembly for biomedical question answering across literature, knowledge bases, and biological atlases

**DOI:** 10.1093/bioinformatics/btag795

**Published:** 2026-10-23

**Authors:** Meng Xiao, Chuan Qin, Jinmiao Chen, Yihang Cheng, Yuanchun Zhou, Hengshu Zhu

**Affiliations:** Computer Network Information Center, Chinese Academy of Sciences, Beijing 100180, China; DUKE-NUS Medical School, National University of Singapore, 169857, Singapore; Computer Network Information Center, Chinese Academy of Sciences, Beijing 100180, China; University of Chinese Academy of Sciences, 101408, Beijing, China; DUKE-NUS Medical School, National University of Singapore, 169857, Singapore; Bioinformatics Institute, Agency for Science, Technology and Research, 138671, Singapore; Computer Network Information Center, Chinese Academy of Sciences, Beijing 100180, China; Computer Network Information Center, Chinese Academy of Sciences, Beijing 100180, China; University of Chinese Academy of Sciences, 101408, Beijing, China; Computer Network Information Center, Chinese Academy of Sciences, Beijing 100180, China; University of Chinese Academy of Sciences, 101408, Beijing, China

## Abstract

**Motivation:**

Biomedical question answering often requires evidence beyond topically retrieved literature, including gene alias resolution, database identifier normalization, and atlas-derived biological measurements. However, existing retrieval-augmented generation (RAG) systems typically follow a fixed workflow and lack an explicit mechanism for deciding when retrieved text is sufficient, when biomedical entity mapping is needed, or when executable evidence assembly over structured measurements should be invoked. This motivates a substrate-aware large language model (LLM) harness that selectively assembles sufficient evidence by combining literature retrieval, biomedical entity mapping, and access to atlas-derived measurements.

**Results:**

We introduce BioHarness, an LLM harness for staged biomedical evidence assembly across literature retrieval, biomedical entity-mapping resources, and atlas-derived structured measurements. Across 22 123 biomedical QA items spanning seven answer formats, BioHarness improves the pooled score from 62.2 to 67.2 over the strongest baseline. Ablations, case studies, and backbone-scaling analyses show that these gains arise from repairing evidence-substrate mismatches through reranking, entity grounding, and structured measurement access, rather than from indiscriminately invoking more reasoning steps, retrieving additional literature, or relying on a particular answer-model scale. Comparisons with the frontier models and harness show that BioHarness, despite using an open-weight backbone with only 3B active parameters, approaches frontier-level performance on selected literature QA tasks and outperforms frontier harnesses without web access on biomedical identifier and locus lookup.

**Availability and implementation:**

The source code, reproducible benchmarking scripts, and processed benchmark data required to reproduce all results in this study are publicly available under an open-source license, together with the exat configuration, model versions, and decoding settings used in our experiments. In addition, we developed a prototype web demo (https://xcompass.scimatrix.cn/) that illustrates how BioHarness can be used in an interactive biomedical QA setting.

## 1 Introduction

Biomedical question answering with large language models (Luo *et al.* 2025, Zhou *et al.* 2025, Zhang *et al.* 2026) can require more than literature retrieval. A biomedical question may require an answer-bearing literature statement (Jin *et al.* 2022, 2023), canonical entity grounding behind an ambiguous surface mention (Gyori *et al.* 2022, Wei *et al.* 2024), or a measured expression pattern stored in a bulk expression resource, tissue atlas, or single-cell atlas (Consortium *et al.* 2022, Li *et al.* 2022, George *et al.* 2024, CZI Cell Science Program *et al*. 2025). We use *substrate-aware* to describe coordination across these resource types and their different access operations: text retrieval over literature, entity mapping through database lookup, and queries over structured atlas measurements. Fixed retrieve-then-generate pipelines can therefore fail through evidence-substrate mismatch: topically relevant text may not contain answer-supporting evidence, surface names may not resolve to the correct biomedical identity, and measured biological values may be absent from narrative abstracts even when they are available in structured resources (illustrated in Fig. 1, available as supplementary data at *Bioinformatics* online). BioHarness addresses this mismatch through the staged evidence assembly pipeline shown in Fig. 1.

**Figure 1:**
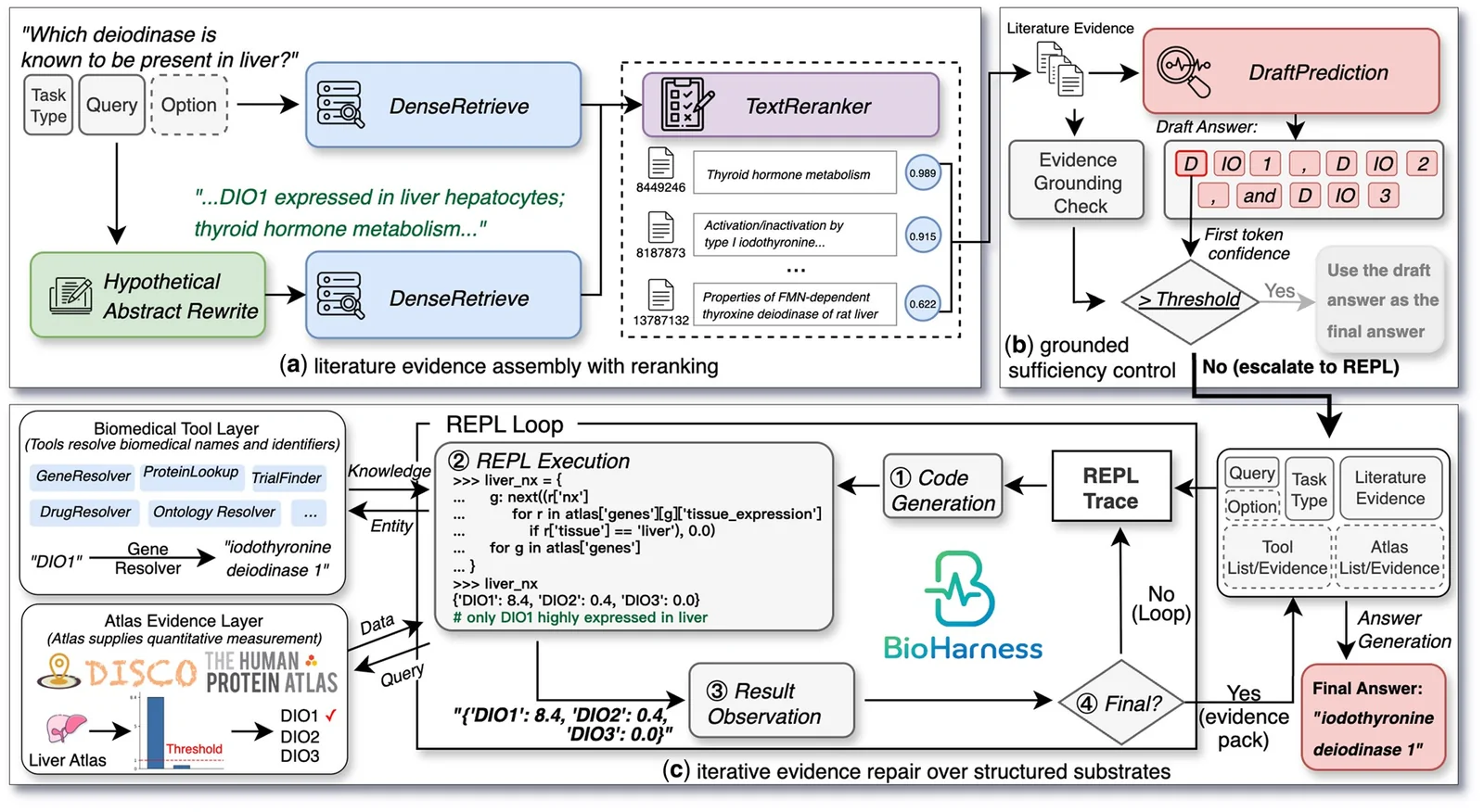
BioHarness implements a substrate-aware biomedical evidence assembly pipeline consisting of (a) literature evidence assembly with reranking, (b) grounded evidence sufficiency control, and (c) executable evidence assembly over biomedical tools and atlas-derived structured measurements, followed by a task-constrained answer generation head.

Existing biomedical RAG systems improve individual parts of this process, but rarely treat evidence-substrate selection as an explicit control problem. Text-centered methods, including dense retrieval, query expansion, reranking, and reflective evidence filtering, improve the recall or ranking of relevant passages, but still assume that the answer-supporting evidence can be recovered from retrieved text (Karpukhin *et al.* 2020, Lewis *et al.* 2020, Asai *et al.* 2023, Gao *et al.* 2023, Yu *et al.* 2024a, 2024b). Biomedical tool-augmented and knowledge-graph-based methods incorporate structured resources or entity-relation context, yet these resources are often attached as fixed preprocessing, auxiliary context, or graph summaries rather than invoked according to evidence sufficiency (Edge *et al.* 2024, Jin *et al.* 2024, Matsumoto *et al.* 2024, Soman *et al.* 2024, Wang *et al.* 2024, Li *et al.* 2025). Agentic retrieval and reasoning frameworks can further interleave search, tool use, and multi-step inference, but indiscriminate execution increases costs and may still consult the wrong evidence source when the question requires canonical entity grounding or database-native measurements (Yao *et al.* 2022, Shinn *et al.* 2023, Trivedi *et al.* 2023). Thus, the central problem is not simply how to retrieve more passages, add more tools, or reason for more steps, but how to decide which evidence source suffices for a given biomedical question.

To address this limitation, we introduce BioHarness, a substrate-aware LLM harness for staged biomedical evidence assembly and question answering. Following recent usage for LLM agents (Guo *et al.* 2026), we use *LLM harness* to denote the inference-time software layer around a language model that assembles its context, invokes models and tools, maintains intermediate state, and decides when to stop, without updating model weights. BioHarness specializes this layer for biomedical QA: for each question, it decides what evidence to assemble, which resources to query, and whether the assembled evidence is sufficient to answer. When the retrieved evidence is uncertain, weakly grounded, or mismatched to the question requirement, BioHarness escalates to iterative evidence repair in a read–eval–print loop (REPL) environment, which executes code, returns results, and preserves intermediate state across steps. Within this environment, the LLM can inspect passages, invoke biomedical tools for entity grounding, access router-gated atlas evidence, and assemble a compact evidence state for final answer generation. A task-constrained output head finally normalizes the answer to the required format, allowing the same harness to support yes/no, multiple-choice, factoid, list, summary, and expression-style questions.

Our contributions are threefold. First, we identify evidence-substrate mismatch as a central failure mode in biomedical RAG, showing that failures can arise not only from insufficient retrieval but also from applying textual retrieval to questions that require entity grounding or structured biological measurements. Second, we propose BioHarness, a substrate-aware LLM harness that coordinates literature retrieval, biomedical entity mapping, and atlas measurement access through dual evidence retrieval, question-conditioned reranking, cascade control, biomedical tools, router-gated atlas access, REPL-style iterative evidence repair, and task-constrained answer generation. Third, we conduct an extensive controlled analysis across heterogeneous biomedical QA datasets, component ablations, efficiency settings, backbone scales, and mechanistic case studies, demonstrating that substrate-aware evidence assembly improves biomedical QA beyond fixed retrieval, query-expansion, reflective, tool-augmented, and graph-based RAG pipelines.

## 2 Materials and methods

As shown in Fig. 1 and Algorithm 1, available as supplementary data at *Bioinformatics* online, BioHarness implements a substrate-aware biomedical evidence assembly pipeline that first attempts to answer from literature evidence and escalates only when additional evidence are needed. When the literature context is uncertain, weakly grounded, or substrate-mismatched, REPL-style iterative evidence repair incorporates biomedical tool outputs and atlas-derived measurement records to construct a sufficient evidence state.

### 2.1 Literature evidence assembly

The literature evidence component constructs an initial evidence context from a biomedical literature corpus of PubMed abstracts and full text. This component addresses a basic limitation of single-query dense retrieval: top-ranked abstracts may be topically related to the question, but still miss the specific answer-bearing evidence needed by the downstream task. Within the literature substrate, BioHarness combines complementary retrieval views with question-conditioned reranking to improve recall while preserving answer relevance.

As shown in Fig. 1a, the first retrieval view uses the original question, preserving literal wording and entity-level precision. The second retrieval view follows the hypothetical-document retrieval principle (Gao *et al.* 2023): the LLM rewrites the question as a short PubMed-style hypothetical abstract (the full rewrite prompt is provided in Note 2, available as supplementary data at *Bioinformatics* online), thereby moving the retrieval query closer to a plausible biomedical evidence context. The retrieved candidates from active retrieval views are merged, de-duplicated by document identifier when available, and reranked against the original question. This reranking step mitigates hypothesis drift from the rewritten query while retaining its recall benefit.

The resulting Literature Evidence context is passed to the cascade controller as the initial evidence substrate. This component is not intended to solve all biomedical evidence needs through retrieval alone: when the primary need is canonical entity grounding or structured biological measurement, the retrieved literature is retained as a lightweight context and the unresolved evidence need is delegated to downstream structured substrates. Literature evidence assembly thus serves as the first substrate in a staged process, rather than as a universal retrieval solution.

### 2.2 Grounded evidence sufficiency control

After constructing the Literature Evidence context, BioHarness forms a *literature evidence* context by merging reranked literature with lightweight tool-derived grounding signals from a biomedical tool precall. The grounded sufficiency control then decides whether this fast evidence is sufficient for answer generation or whether the query should be escalated to iterative evidence repair. As shown in Fig. 1b and Algorithm 1, available as supplementary data at *Bioinformatics* online, the controller generates a task-constrained draft answer $a^{\left(0\right)}$ from the fast evidence context $E_{\text{fast}}$ and derives two complementary routing signals.

The first signal is a confidence proxy c=exp(ℓ), where ℓ is the log-probability of the first generated token. This signal is used only as a lightweight routing heuristic, not as a calibrated estimate of answer correctness (Kadavath *et al.* 2022). The second signal is an evidence-grounding score *e*, which estimates whether the draft answer is supported by the assembled evidence. For text-grounded answers, this check compares normalized answer strings or content tokens against retrieved titles and abstracts; for entity-centric questions, it also incorporates tool-derived grounding signals such as resolved symbols, aliases, identifiers, or database records.

The controller is answer-form aware rather than benchmark-specific. For closed-form outputs, such as yes/no answers or option letters, the confidence proxy is often sufficient when the answer is explicitly supported by the fast evidence context. For open-form outputs, such as factoid entities, lists, summaries, or expression sets, confidence alone is less reliable because an answer may be locally fluent but incomplete, unsupported, or mismatched to the required evidence substrate. In these cases, grounding provides the decisive safeguard: BioHarness escalates when the draft is empty, low-confidence, insufficiently grounded, or inconsistent with the evidence substrate required by the question. The answer-form policy and thresholds are fixed on a development split before final evaluation and are detailed in Note 6, available as supplementary data at *Bioinformatics* online.

If escalation is not triggered, the draft answer is returned through the fast path. Otherwise, the query enters REPL-style iterative evidence repair, where biomedical tools and router-gated atlas records are initialized as structured substrates for stateful evidence assembly. This policy preserves confident fast path answers when reranked literature and tool-derived grounding are already sufficient, while reserving more expensive iterative assembly for cases that require additional entity grounding, evidence reconciliation, or structured measurement access. The full controller and assembly procedures are provided in Algorithms 1 and 2, available as supplementary data at *Bioinformatics* online.

### 2.3 Structured biomedical evidence layers

When literature evidence is insufficient, BioHarness can access two structured biomedical evidence layers: the Biomedical Tool Layer and the Atlas Evidence Layer (Tool schemas, atlas sources, router criteria, and coverage are detailed in Notes 3 and 4, available as supplementary data at *Bioinformatics* online). Together, they provide access patterns that are difficult to recover through dense retrieval alone. The Biomedical Tool Layer supports entity-level grounding, including gene-symbol normalization, alias and deprecated-symbol resolution, identifier lookup, ontology traversal, and lightweight database-style checks. These operations are treated as operations that link surface mentions to canonical biomedical entities and identifiers. Their outputs can directly answer identity or identifier questions and help associate retrieved information with the intended entity.

The Atlas Evidence Layer supports measurement-level grounding through a read-only atlas namespace. Atlas access is controlled by a router that converts atlas-relevant questions into an atlas query specification, including candidate genes, tissues or cell types, measurement fields, and optional operations such as filtering or thresholding. Only routed cases fetch the corresponding atlas entries before REPL execution. The fetched atlas object is represented as structured measurement evidence, containing gene metadata, tissue-expression records, cell-type annotations, quantitative values, and provenance fields when available. During REPL execution, these structured records can be inspected, filtered, aggregated, or thresholded through deterministic operations. The two substrates are deliberately separated because biomedical tools resolve names, identifiers, aliases, and relations, whereas atlas access provides biological measurements.

### 2.4 Iterative evidence repair

When the controller rejects the fast evidence path, BioHarness enters an REPL-style iterative evidence-assembly loop. As detailed in Algorithm 2, available as supplementary data at *Bioinformatics* online, the loop is not used as an unconstrained reasoning chain. Instead, it provides an executable workspace for substrate-aware evidence assembly: the LLM can inspect retrieved passages, invoke read-only biomedical tools, access router-gated atlas evidence, and assemble a compact evidence state through executable operations.

This design targets two common needs after literature retrieval. First, some questions require symbolic biomedical grounding rather than additional text retrieval. Deprecated aliases, surface-form ambiguity, and missing identifiers are better resolved by gene normalization and database lookup tools than by repeatedly searching the literature corpus. Second, atlas-derived evidence is often structured and numeric rather than narrative. Expression patterns, tissue distributions, and cell-type measurements naturally require executable operations such as filtering rows, applying thresholds, aggregating values, or constructing entity sets. A REPL-style interface therefore matches the access pattern of structured atlas evidence more closely than a pure retrieve-then-generate pipeline.

The evidence trace *h* records the action–observation history and is later compressed into an evidence pack for final answer generation. This gives the harness two properties that ordinary retrieve-then-generate pipelines lack: observations are stateful, so intermediate outputs such as resolved symbols, entity sets, and filtered atlas measurements remain available for later operations; and execution is inspectable, so the trace exposes whether an assembly came from entity normalization, database lookup, or atlas filtering, making evidence assembly both substrate-aware and auditable.

### 2.5 Task-constrained answer generation

After either the fast evidence path or REPL-style iterative evidence repair, BioHarness produces the final answer through a task-constrained output head. For fast-path cases, the draft answer is returned when the controller determines that it is sufficiently confident and grounded in the fast evidence context. For escalated cases, the REPL trace is compressed into an evidence pack that includes retrieved literature snippets, resolved biomedical entities, tool observations, atlas-derived measurement records, and, when available, provenance information. The output head then converts this evidence pack into an answer compatible with the task specification and answer options. This separates evidence assembly from answer formatting: rather than relying on unconstrained free-form generation, the head constrains the response to the required output space (closed-set options, normalized entity spans or sets, or concise evidence-grounded summaries), ensuring that heterogeneous evidence traces from literature, biomedical tools, and atlas-derived measurements are converted into outputs compatible with the evaluation protocol.

### 2.6 Implementation details

We evaluate all methods with the same answer backbone [Qwen3.5-35B-A3B (https://huggingface.co/Qwen/Qwen3.5-35B-A3B)] on a unified biomedical QA benchmark containing 22 123 questions from ten public datasets, normalized into a common JSONL schema and evaluation protocol (https://huggingface.co/datasets/Shaow/BioHarness_Eval). The benchmark covers heterogeneous answer formats, including yes/no questions, single-answer multiple-choice questions, multi-answer choice questions, short factoid answers, list-style answers, summaries, and structured expression outputs. The benchmark covers clinical reasoning datasets, including MedMCQA (Pal *et al.* 2022), MedQA-US/TW/CN (Jin *et al.* 2020), and MedXpertQA-Text (Zuo *et al.* 2025); literature-grounded datasets, including BioASQ (Krithara *et al.* 2023), PubMedQA (Jin *et al.* 2019), and LitQA2 (Laurent *et al.* 2024); and genomics-oriented datasets, including GeneTuring (Shang *et al.* 2025) and SciHorizon-Gene (Qin *et al.* 2025, Huang *et al.* 2026). Each item is scored using a task-specific matching function and all scores are reported on a 0–100 scale. We use exact match for yes/no and single-choice questions, set-F1 for multi-answer, list-style, and expression-style questions, token-level F1 for factoid questions (Rajpurkar *et al.* 2016), and ROUGE-L F1 for summary questions (Lin 2004); dataset-level and overall scores are computed as pooled item-level averages. All baselines in Table 1 are evaluated under the same controlled platform, sharing the processed corpus, dense retriever, answer backbone, constrained decoding strategy, and evaluation pipeline, with reranking applied only in the corresponding reranking settings. Detailed baseline descriptions, dataset normalization procedures, gold-evidence availability, and formal metric definitions are provided in Note 1, available as supplementary data at *Bioinformatics* online.

## 3 Results

### 3.1 Main results on the unified biomedical QA benchmark

Table 1 reports the main comparison on ten biomedical QA benchmarks. BioHarness achieves the highest pooled score, improving over the strongest non-oracle baseline, HyDE, from 62.2 to 67.2, while using a moderate compute budget. It obtains the highest score on nine of the ten benchmarks; the exception is MedQA-TW, where Self-RAG scores slightly higher. The largest gains appear in benchmarks that require precise biomedical grounding: BioHarness substantially improves GeneTuring and SciHorizon, suggesting that adaptive evidence control is more effective than fixed retrieval or tool-use strategies. Besides, MedXpertQA-Text mainly stresses expert-level clinical reasoning, where BioHarness’s gains are small.

**Table 1:** Main results on ten biomedical QA benchmarks with unified backbone Qwen3.5-35B-A3B.

Method	PubMedQA	BioASQ	GeneTuring	SciHorizon	MedMCQA	MedQA-US	MedQA-TW	MedQA-CN	MedXpertQA-Text	LitQA2	Overall
No-context	50.6	45.4	7.9	42.1	72.7	82.0	89.1	89.3	35.1	38.7	58.9
w/Golden Context^a^	78.0	66.4	–	–	–	–	–	–	–	94.8	–
DenseRAG (Karpukhin *et al.* 2020)	75.4	47.2	8.9	43.3	73.1	84.0	89.0	89.4	33.9	45.9	60.2
HyDE (Gao *et al.* 2023)	75.4	53.3	10.6	48.1	72.9	84.1	88.5	88.8	36.3	44.9	62.2
DenseRAG + Rerank (Karpukhin *et al.* 2020)	75.9	50.1	9.6	45.4	73.5	82.6	89.3	89.0	33.8	58.1	61.1
Chain-of-Note (Yu *et al.* 2024a)	73.4	49.9	7.7	44.8	71.9	82.1	88.0	88.9	33.5	46.4	60.3
FLARE (Jiang *et al.* 2023)	72.4	39.2	9.7	51.7	63.4	78.1	74.7	77.9	27.9	17.3	53.6
Self-RAG (Asai *et al.* 2023)	74.0	48.7	9.1	45.1	73.1	81.9	**89.5**	89.2	36.9	47.4	60.9
IRCoT (Trivedi *et al.* 2023)	76.0	53.8	8.7	46.4	73.0	82.8	88.1	88.8	31.9	55.1	61.6
GraphRAG (Edge *et al.* 2024)	50.8	44.9	8.7	44.7	72.0	82.2	87.8	89.0	33.0	20.9	58.5
LightRAG (Guo *et al.* 2024)	57.0	44.6	9.0	44.2	71.6	82.2	88.3	88.8	31.4	14.1	58.2
PathRAG (Chen *et al.* 2026)	49.8	43.3	7.3	44.6	66.0	81.9	85.6	83.4	32.2	16.9	55.7
BiomedRAG (Li *et al.* 2025)	75.0	47.8	9.5	44.8	72.9	82.4	88.3	89.1	34.9	56.3	60.5
BioRAG (Wang *et al.* 2024)	47.6	37.1	21.4	50.3	72.9	82.2	89.2	89.0	34.4	36.8	58.7
GeneGPT (Jin *et al.* 2024)	75.4	47.0	34.5	50.6	72.8	83.2	88.5	89.0	33.4	46.9	62.1
BioHarness	**76.4**	**54.1**	**54.6**	**60.3**	**74.9**	**85.9**	89.3	**89.7**	**37.1**	**61.8**	**67.2**

a *Golden Context* uses dataset-provided gold evidence when available and is reported as an oracle evidence upper bound; it is excluded from best/second-best highlighting. Among non-oracle methods, the best and second-best results are shown in **bold** and underlined, respectively; ties are marked accordingly.

The comparison also shows that stronger retrieval alone is insufficient. Dense retrieval, reranking, reflective retrieval, and graph-based retrieval each improve some settings, but their gains remain uneven across the benchmark. Similarly, biomedical tool-augmented methods such as BioRAG and GeneGPT improve entity-centric tasks, but do not consistently generalize to the full benchmark. These results suggest that biomedical QA benefits more from adaptive control over retrieval, reranking, structured-resource invocation, and evidence repair than from any single fixed retrieval architecture.

The “w/Golden Context” setting provides an evidence upper bound only for PubMedQA, BioASQ, and LitQA2, where dataset-provided gold evidence is available. BioHarness approaches the oracle on PubMedQA, whereas larger gaps remain on BioASQ and LitQA2. Because the oracle removes the retrieval problem, these gaps locate the remaining bottleneck in evidence retrieval and assembly. The oracle’s own shortfall reflects limits of downstream synthesis, answer normalization, and task-specific scoring that better evidence alone does not remove. In general, the results support the central design of BioHarness: accurate biomedical question answering requires not only retrieving relevant documents, but also assembling and processing evidence according to the question type and the available biomedical resources.

### 3.2 Efficiency of grounded cascade control

Figure 2 summarizes the score–compute tradeoff on the full benchmark. We measure median end-to-end latency per question, average token usage, and average LLM calls under the same evaluation setting as Table 1. For graph-based retrieval methods, latency includes query-time graph construction and reasoning, so the reported operating point reflects a single-shot deployment scenario without cross-query caching.

**Figure 2:**
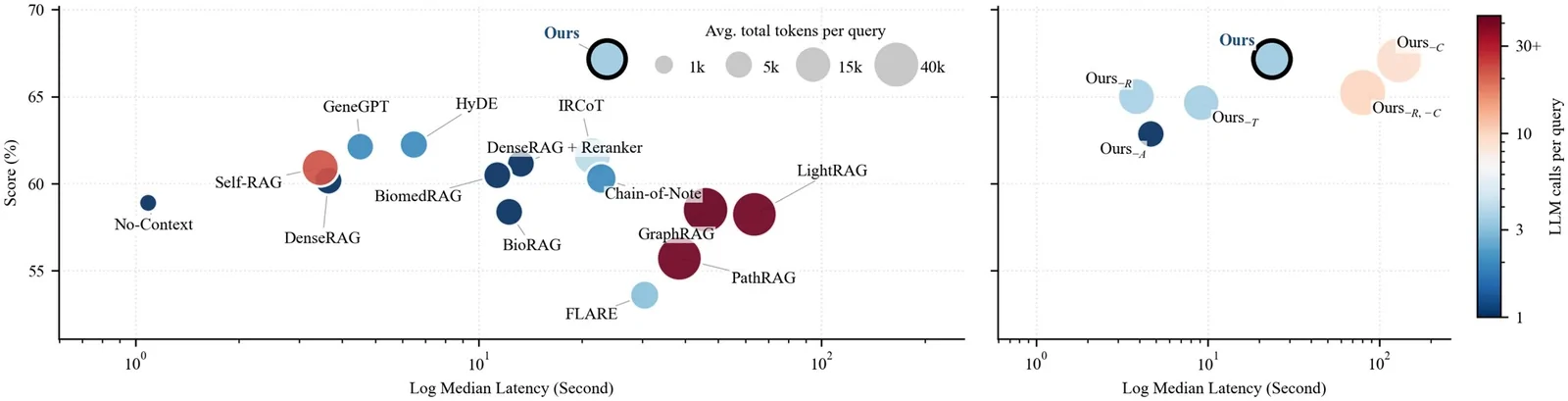
Score–efficiency comparison on the unified 22 123-question benchmark. The left panel compares BioHarness with all baselines (all with Qwen3.5-35B-A3B), and the right panel shows component ablations of BioHarness. Here, Ours${\;}_{-A}$ disables REPL iterative reasoning escalation while retaining fast-path tool-derived grounding; Ours${\;}_{-C}$ removes cascade routing and forces all questions into the agent route; Ours${\;}_{-T}$ disables the Biomedical Tool Layer; Ours${\;}_{-R}$ removes question-conditioned reranking; and Ours${\;}_{-R,-C}$ disables both the cascade routing and question-conditioned reranking component. Each point reports the pooled score against median per-question latency on a log-scaled horizontal axis. Bubble area denotes the average total token count per query, and color denotes the average number of LLM calls per query.

BioHarness lies on the empirical efficient frontier. It achieves the highest pooled score, improving over the strongest non-oracle baseline, HyDE, while using a moderate compute budget. In contrast, several methods that spend substantially more computation do not obtain comparable scores. GraphRAG, PathRAG, and LightRAG require many LLM calls and large token budgets because they construct or traverse query-local graph structures, yet their scores remain below strong dense, query-augmentation, or reflective retrieval baselines. This indicates that the gain of BioHarness is not simply due to spending more inference compute, but to allocating computation according to evidence sufficiency.

The ablation panel further clarifies the role of cascade routing. Removing cascade control, BioHarness${\;}_{-C}$, forces all questions into the agent route and gives nearly the same pooled score as the full pipeline, but shifts markedly toward the high-latency and high-token regime. The full grounded cascade recovers the same accuracy level with much lower latency and fewer tokens by preserving confident fast-path answers and escalating only uncertain, weakly grounded, or substrate mismatched cases. Conversely, disabling REPL iterative reasoning escalation, BioHarness${\;}_{-A}$, yields a cheaper but substantially lower-scoring configuration, confirming that iterative repair is useful when needed but should not be applied indiscriminately.

Overall, the efficiency analysis supports the central design choice of BioHarness: literature retrieval, question-conditioned reranking, biomedical tools, atlas access, and REPL-style repair should be treated as a staged evidence-allocation policy rather than as uniformly applied modules. The cascade preserves the score benefit of iterative repair while avoiding unnecessary agent execution for questions that can already be resolved by the fast path with reranked literature and tool-derived grounding. Similarly, atlas access is router-gated rather than globally enabled: only a small fraction of benchmark queries are routed to the Atlas Evidence Layer, indicating that structured measurement queries are rare at the benchmark level but require a dedicated substrate when they arise. This behavior supports the role of BioHarness as a substrate-aware harness that selectively allocates computation and switches evidence sources, instead of applying all retrieval, tool, and atlas operations to every question.

### 3.3 Backbone scaling analysis

To examine whether the gains of BioHarness depend on a particular answer-model scale, we reevaluated BioHarness and four representative backbone-swappable baselines (DenseRAG, HyDE, Self-RAG, and BioRAG) across seven Qwen3.5 backbones ranging from 0.8B to 122B-A10B, on a deterministic 10% subsample of the unified benchmark (Fig. 3). These baselines were selected because they are backbone-swappable under our controlled platform and together cover representative retrieval paradigms, namely dense retrieval, query augmentation, reflective retrieval, and biomedical tool-augmented retrieval. BioHarness obtains the highest pooled score at every tested backbone size: the margin is small at 0.8B, where all methods remain in a low-score regime, but becomes clearer from 2B onward, and larger backbones improve all methods without removing the gap. A paired item-level analysis confirms this trend, with BioHarness maintaining a positive win–loss balance against all four baselines at every backbone size, indicating that the gain reflects consistent item-level improvements rather than a few high-scoring outliers. This analysis supports the claim that the gains of BioHarness do not arise merely from using a particular answer backbone, but from improving the evidence available before generation through literature evidence ranking, biomedical entity grounding, and executable evidence repair.

**Figure 3:**
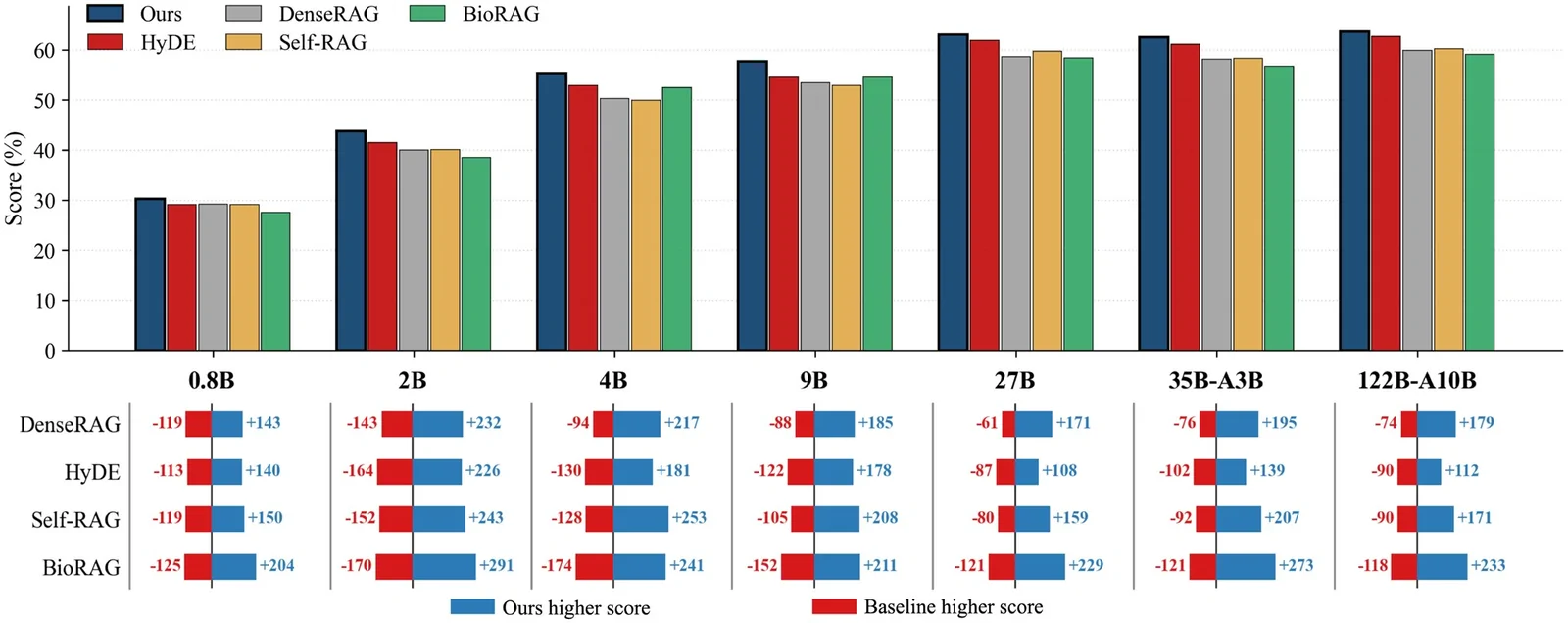
Backbone scaling analysis of BioHarness on 10% subsample of 10% subsample of the unified benchmark. The top panel reports the pooled score across seven Qwen3.5 backbones ranging from 0.8B to 122B-A10B. The bottom panel shows paired item-level comparisons between BioHarness and representative baselines; blue bars denote items where BioHarness obtains a higher item-level score than the baseline, and red bars denote the converse.

### 3.4 Component ablation studies

Figure 4 analyzes the contributions of the major retrieval, control, repair, and atlas components of BioHarness. Disabling REPL iterative reasoning escalation (Ours${\;}_{-A}$) yields the largest pooled degradation in the current analysis, reducing the score from 67.2 to 62.7. Disabling the Biomedical Tool Layer (Ours${\;}_{-T}$) reduces the score to 64.7, while removing question-conditioned reranking (Ours${\;}_{-R}$) reduces it to 65.0. Removing both reranking and cascade control (Ours${\;}_{-R,-C}$) yields 65.1. Removing cascade control alone (Ours${\;}_{-C}$), which forces all questions into the agent route, gives a similar pooled score of 67.1 but changes the error profile. This indicates that the grounded cascade is not only a cost-saving mechanism; it also protects already-grounded fast-path answers from unnecessary agent-side rewriting or over-processing.

**Figure 4:**
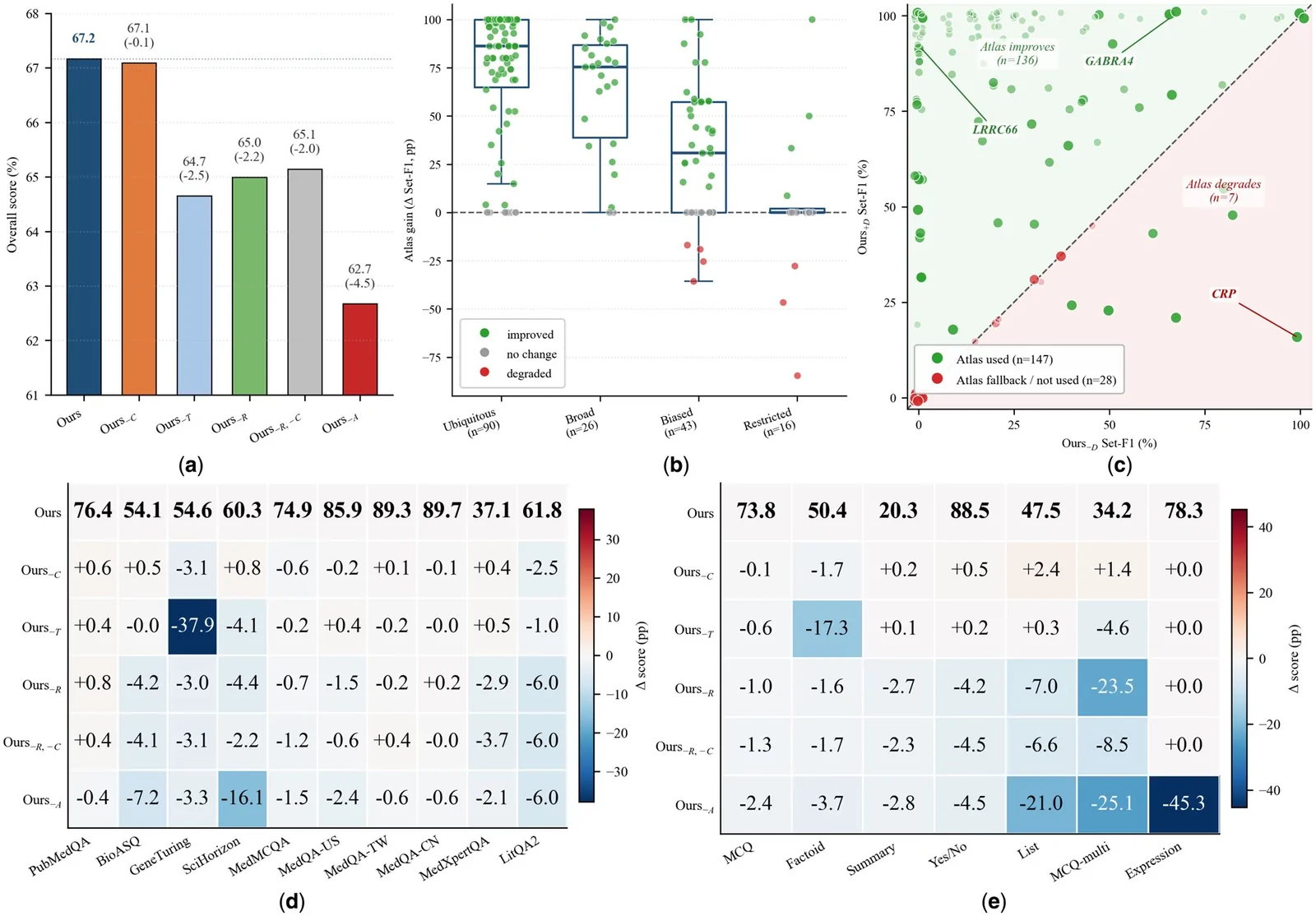
Component ablation and atlas-routed evidence analysis of BioHarness (Qwen3.5-35B-A3B). (a) Pooled overall score under major component ablations on the unified benchmark. (b) Distribution of atlas-induced Set-F1 changes on atlas-relevant expression questions, grouped by tissue-specificity class. (c) Paired comparison between our model and Ours${\;}_{-D}$ on atlas-routed cases, where Ours${\;}_{-D}$ disables atlas-derived measurement access; points above the diagonal indicate cases improved by atlas evidence. (d) Per-dataset score changes relative to the full model. (e) Per-task score changes relative to the full model.

Panels (d) and (e) show where these changes occur. At the dataset level, disabling the Biomedical Tool Layer causes the largest loss on GeneTuring, consistent with the importance of tool-based alias resolution, canonical entity grounding, and identifier normalization for gene-centric questions. By contrast, removing cascade control causes only a small pooled change, but it slightly degrades GeneTuring, where many questions can already be answered correctly through fast-path retrieval plus tool-derived grounding. This supports the intended role of the grounded gate: it escalates uncertain or substrate-mismatched cases while preserving confident fast-path answers when additional agent reasoning is more likely to perturb than repair the answer. Disabling REPL iterative escalation mainly affects SciHorizon and BioASQ, indicating that iterative repair is most useful when the model must recover or synthesize evidence beyond a single retrieved passage. At the task level, this degradation is most pronounced for expression, multi-answer choice, and list-style questions, which often require evidence aggregation or repeated checking. Removing reranking also hurts BioASQ and SciHorizon, and particularly affects list and multi-answer settings, supporting its role in selecting answer-bearing evidence from topically similar biomedical abstracts.

Panels (b) and (c) isolate the Atlas Evidence Layer, which is router-gated and therefore not well summarized by a single pooled benchmark score. Panel (b) shows that atlas-derived measurements provide substantial gains for many atlas-relevant expression questions, especially when tissue-level expression patterns are broad or under-represented in the retrieved literature. Panel (c) further compares BioHarness with Ours${\;}_{-D}$ on atlas-routed cases. Most points lie above the diagonal, indicating that atlas evidence improves the routed examples, while degradation is rare and concentrated in a small number of cases.

Qualitatively, the paired atlas analysis reveals two dominant improvement modes and one residual failure mode. For genes with under-reported literature evidence, atlas measurements recover missing expression sites, as in GABRA4, where the atlas-disabled variant retrieves only brain while atlas evidence completes the ground truth with cardiac expression. For sparsely characterized genes, atlas evidence can be decisive: LRRC66 receives no usable tissue assignment from literature retrieval, whereas atlas-derived measurements recover a gastrointestinal expression pattern with high Set-F1. The main degradation mode occurs when the literature already provides a precise restricted answer, but the atlas introduces broad low-level off-target signals, as in CRP, where over-trusting noisy atlas breadth expands the answer beyond liver and reduces Set-F1. Thus, atlas access is most useful when literature priors are incomplete or weak, but requires conservative filtering when structured measurements are broader than the benchmark target.

Overall, the ablation results support the substrate-aware design of BioHarness. Literature reranking improves answer-bearing evidence selection, biomedical tools repair entity-level grounding failures, REPL iterative escalation repairs unresolved cases beyond the fast path, and atlas-derived measurements supply a targeted substrate for measurement-centric questions.

### 3.5 Frontier models and harness comparison

To separate the contributions of backbone strength, harness, and evidence access, we compare BioHarness with claude-opus-5 and gpt-5.6-sol in three settings (Fig. 5). Standalone models are queried through OpenRouter, and the harness settings use the default settings in Claude Code and Codex CLI, respectively, with or without web access. With web access, BioHarness’s answer stage receives the pages fetched by Codex.

**Figure 5:**
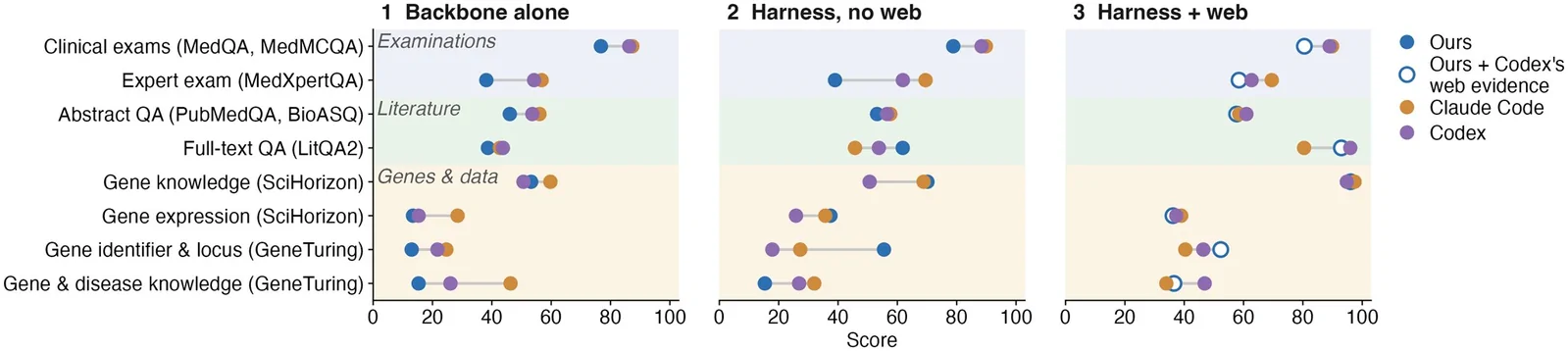
Frontier coding harnesses and BioHarness (Qwen3.5-35B-A3B) compared in three stages, by task type. Every point is computed on 5% sample of the nine benchmarks plus all 199 LitQA2 items. LitQA2 scores in the web setting exclude 13 items for which search returned a public copy of the benchmark.

Backbone strength and evidence access help with different kinds of questions. In questions whose answers must be looked up rather than recalled, BioHarness gains 18–43 points over its backbone alone on LitQA2, SciHorizon gene knowledge, and GeneTuring identifier and locus lookup, several times the gain from switching to a frontier backbone, and exceeds both frontier harnesses without web access on LitQA2 and identifier and locus lookup. On clinical and expert-level examinations, the pattern reverses: the frontier backbones remain stronger, and all three harnesses without web access improve little. With web access, the frontier harnesses overtake BioHarness on literature questions but remain below it on identifier and locus lookup. Given the same Codex-fetched pages, BioHarness’s answer stage recovers most of Codex’s LitQA2 score with its Qwen backbone, suggesting that the frontier advantage on this task comes mainly from evidence access rather than backbone strength.

Together with the tool and atlas ablations, these results suggest that, for questions whose answers must be looked up, the evidence matters more than the backbone strength. Further, BioHarness provides this access with an open-weight backbone with 3B active parameters, much lower than the frontier harnesses, and without external model APIs or open-web access. Because the evidence it relies on resides in its literature index, tools, and atlas rather than in model parameters, it can be updated by indexing new literature or adding resources, without retraining the backbone.

### 3.6 Mechanistic case analyses

We also provide three representative cases in Note 8, available as supplementary data at *Bioinformatics* online, to illustrate how BioHarness resolves distinct failure modes through substrate-aware control. The first case shows that cascade routing avoids unnecessary agent execution: for a PubMedQA yes/no question whose answer is already explicitly supported by the top-ranked passage, BioHarness returns the same grounded answer as the forced-agent variant (BioHarness${\;}_{-C}$) while reducing latency by 7.4×. The second case shows the role of biomedical entity grounding: for the deprecated gene alias AGTIL, literature retrieval fails to bridge the alias to the official symbol, whereas the Biomedical Tool Layer resolves AGTIL→ASIP. The third case shows the need for atlas-derived measurement evidence: for the tissue-expression question on FDXR, retrieved abstracts discuss disease mechanisms rather than tissue-level expression, while atlas access recovers the expression set and improves Set-F1 from 0.0 to 80.0. Full annotated evidence traces are provided in Note 8 and Figs 3–5, available as supplementary data at *Bioinformatics* online.

## 4 Discussion

This work highlights evidence-substrate mismatch as a key failure mode in biomedical RAG. BioHarness addresses this issue by coordinating literature retrieval, biomedical entity tools, and atlas-derived structured measurements within a staged evidence assembly framework. The results indicate that selective escalation is more effective than indiscriminately invoking multi-step reasoning, because additional execution is useful when it repairs weakly grounded, uncertain, or substrate-mismatched evidence. This suggests that future biomedical QA systems should be designed as substrate-aware harnesses that select among these resources according to the evidence a question requires, rather than treating all evidence as retrievable text. Several limitations remain. First, the evaluation uses predefined QA benchmarks rather than open-ended research workflows, and atlas access currently covers only bulk and single-cell expression measurements, so the routing frequencies observed here may not transfer to everyday bioinformatics work. Second, the cascade controller depends on a first-token confidence proxy and requires token log-probabilities, which API-only models may not expose. The stateful REPL provides a basis for extending the harness to further analysis scenarios, such as routing expression-comparison questions to differential-expression workflows. This ability requires additional implementation and validation and is not evaluated in the present study.

## Supplementary Material

btag795_Supplementary_Data

## Data Availability

The processed benchmark data underlying this article are available at https://doi.org/10.57967/hf/10598, and the source code and benchmarking scripts are available at https://doi.org/10.5281/zenodo.22957864 and https://doi.org/10.5281/zenodo.22957868. The original public datasets integrated into the benchmark are available from their respective sources cited in the text.
