# Supplementary material for: BioHarness: substrate-aware evidence assembly for biomedical question answering across literature, knowledge bases, and biological atlases
Source: Bioinformatics. 2026 Oct 23;42(11):btag795. doi: 10.1093/bioinformatics/btag795 (PMC13739047; doi:10.1093/bioinformatics/btag795)
Supplement: Supplementary file 1 [file btag795_supplementary_data.zip › RAG_RLM_main_BIOINFO__supp.pdf]

Supplementary Material

BioHarness: Substrate-Aware Evidence Assembly for Biomedical Question Answering across Literature, Knowledge Bases, and Biological Atlases

This Supplementary Material collects additional implementation details, algorithm descriptions, prompts, and case traces that are referenced from the main text. Supplementary notes, figures, tables, and algorithms are numbered with an "S" prefix and are cited at the corresponding points of the main text.

Contents

Supplementary Note S1. Baseline, Benchmark datasets, and evaluation metrics 1

- Baseline Methods . . . 1
- Benchmark datasets . . . 2
- Evaluation metrics . . . 2

Supplementary Note S2. Prompts and decoding protocol 3

Supplementary Note S3. Biomedical Tool Layer 3

Supplementary Note S4. Atlas Evidence Layer 3

Supplementary Note S5. Implementation details 4

Supplementary Note S6. Full algorithms and type-conditioned escalation 5

Supplementary Note S7. Substrate overview and retrieval corpus 8

Supplementary Note S8. Mechanistic case studies 9

Supplementary Note S9. Prototype demo 12

Supplementary Note S10. Frontier model and harness comparison 12

Supplementary Note S1. Baseline, Benchmark datasets, and evaluation metrics

Baseline Methods

Following the controlled experimental platform described above, all baselines are evaluated using the same processed corpus, dense retriever, reranker when applicable, answer backbone, constrained decoding strategy, and evaluation pipeline. The compared methods cover retrieval-free, oracle-evidence, dense retrieval, query augmentation, reranking, reflective retrieval, graph-based retrieval, and biomedical tool-augmented retrieval settings.

Retrieval-free and oracle settings. We include *No-Context* as a retrieval-free baseline and *w/ Golden Context* as an oracle-evidence setting using dataset-provided gold evidence when available.
Dense retrieval and query augmentation. We compare with *DenseRAG* (Karpukhin et al., 2020), and *HyDE* (Gao et al., 2023).
Reflective and iterative retrieval. We include *DenseRAG+Rerank*, *IRCoT* (Trivedi et al., 2023), *Chain-of-Note* (Yu et al., 2024), *Self-RAG* (Asai et al., 2023), and *FLARE* (Jiang et al., 2023).
Graph-based retrieval. We compare with *GraphRAG* (Edge et al., 2024), *LightRAG* (Guo et al., 2024), and *PathRAG* (Chen et al., 2026).
Biomedical and tool-augmented retrieval. We further compare with *BiomedRAG* (Li et al., 2025), *BioRAG* (Wang et al., 2024), and *GeneGPT* (Jin et al., 2024).

Benchmark datasets

The unified benchmark integrates ten publicly available biomedical QA datasets into a single JSONL schema and evaluation protocol, comprising 22,123 items across seven task types spanning molecular biology, clinical medicine, and genomics. For the oracle setting, dataset-provided gold evidence is available only for PubMedQA, BioASQ, and LitQA2 (5,418 items).

Each dataset contributes distinct evaluation signals:

- Clinical datasets, including MedMCQA (Pal et al., 2022), MedQA-US, MedQA-TW, MedQA-CN (Jin et al., 2020), and MedXpertQA-Text (Zuo et al., 2025) emphasize diagnostic reasoning and factual recall.
- Literature-based datasets, including BioASQ (Krithara et al., 2023), PubMedQA (Jin et al., 2019), and LitQA2 (Laurent et al., 2024) assess document-grounded inference and summarization.
- Genomics-focused datasets, including GeneTuring (Shang et al., 2025) and SciHorizon-Gene (Qin et al., 2025; Huang et al., 2026), stress domain-specific knowledge, entity normalization, and biological reasoning.

All datasets are normalized into a unified JSONL format, ensuring consistent access to question text, task type, answer options when applicable, and ground-truth annotations.

Evaluation metrics

We evaluate each item using a type-specific matching score and report all scores on a 0–100 scale. For yes/no questions, we use lowercased exact-match accuracy. For single-answer multiple-choice questions, we use exact match on the predicted option letter. For multi-answer choice questions, we compute set-F1 over the predicted and gold option sets. For factoid questions, answers are usually short entities or phrases, so we use token-level F1 (Rajpurkar et al., 2016) after answer normalization, equivalent to unigram-overlap F1. For list-style questions, we compute synonym-aware set-F1 between the predicted and gold answer sets. For summary questions, we use ROUGE-L (Lin, 2004) F1 because it captures longest-common-subsequence overlap and better reflects content preservation in longer free-form answers. For expression-style questions, we compute entity-set F1 over the target entity set.

Let $s_i \in [0, 1]$ denote the type-specific matching score for item i. For a dataset $\mathcal{D}$, the reported dataset score is computed as

$$100 \times \frac{1}{|\mathcal{D}|} \sum_{i \in \mathcal{D}} s_i.$$

The overall score is computed analogously over the full benchmark with size N:

$$100 \times \frac{1}{N} \sum_{i=1}^{N} s_i,$$

Thus, all reported scores are pooled item-level averages of type-specific matching scores.

Supplementary Note S2. Prompts and decoding protocol

All generation-based components share a common backbone with deterministic decoding and identical generation settings across methods and baselines. The hypothetical-abstract rewrite prompt used by the second retrieval view (Section "Literature Evidence Assembly" of the main text) is shown in the box below, where the biomedical question is converted into a hypothetical abstract that is then used as an additional retrieval query. The draft-prediction, final-answer, and REPL code-generation prompts follow the same task-constrained formatting as the output head and are released with the source code[1].

Hypothetical Abstract Rewrite Prompt

```
Please write a short scientific PubMed-style abstract that answers the
question below.  Write in one paragraph, 4--6 sentences, focused on the
biomedical facts directly relevant to the question.

Question:  {question}

Abstract:
```

Supplementary Note S3. Biomedical Tool Layer

The Biomedical Tool Layer exposes read-only functions for entity-level grounding. It supports gene-symbol normalization, alias and deprecated-symbol resolution, identifier lookup, ontology traversal, protein and functional annotation lookup, drug and clinical-trial lookup when routed, and lightweight database-style checks. A lightweight deterministic tool precall extracts structured biomedical entities such as gene symbols, rs identifiers, and DNA sequences, and prepends the resulting tool-derived grounding lines to the fast evidence context. When the cascade escalates, the full routed tool layer is initialized inside the REPL-style iterative assembly environment.

The complete tool inventory is provided in Supplementary Data S1. For clarity, the tools are grouped by access pattern rather than treated as a single homogeneous module: gene, genomics, protein, ontology, drug, and clinical-trial tools support structured biomedical grounding; atlas tools are reported separately as the Atlas Evidence Layer in Supplementary Note S4. This separation ensures that the ablation of the Biomedical Tool Layer refers to entity-level grounding and database-style lookup, whereas atlas-derived measurement access is evaluated separately by the BioHarness$_{-D}$ ablation.

Supplementary Note S4. Atlas Evidence Layer

The Atlas Evidence Layer supplies measurement-level grounding through a read-only atlas namespace. It currently exposes three atlas resource groups: Human Protein Atlas bulk tissue-expression

[1] https://github.com/coco11563/BioHarness

records, DISCO single-cell tissue-level pseudobulk expression, and DISCO cell-type-level expression. The full resource inventory, including species, measurement level, coverage, units, API endpoint, and description, is provided in Supplementary Data S2.

Atlas access is router-gated. For atlas-relevant questions, the router converts the query into an atlas specification containing candidate genes, tissues or cell types, measurement fields, and optional operations such as filtering, aggregation, or thresholding. Only routed cases fetch the corresponding atlas entries before iterative assembly. The fetched object is represented as structured measurement evidence, including gene metadata, tissue-expression records, cell-type annotations, quantitative values, and provenance fields when available. Deterministic operations such as filtering, thresholding, and canonical tissue mapping are then applied before answer generation.

Because benchmark labels use a canonical tissue vocabulary, raw atlas tissues are normalized before evaluation. The raw-to-canonical tissue mapping is provided in Supplementary Data S3, and the organ-level hierarchy and benchmark-vocabulary alignment are provided in Supplementary Data S4. The full DISCO cell-type vocabulary is provided in Supplementary Data S5. Only a small fraction of benchmark queries are routed to the Atlas Evidence Layer, consistent with the rarity of measurement-centric questions at the benchmark level; however, these routed cases require a dedicated structured substrate because their answer-bearing evidence is often stored as database-native measurements rather than narrative statements in the literature.

Supplementary Note S5. Implementation details

Experiments are conducted on a server equipped with eight NVIDIA A800-SXM4-80GB GPUs. For the controlled same-backbone experiments, all Qwen3.5 models are served locally using vLLM[2] with identical decoding settings. Unless otherwise specified, generation-based components use Qwen3.5-35B-A3B[3] as the common backbone, including hypothetical abstract rewriting, draft prediction, REPL-style iterative evidence assembly, and final answer generation. For literature retrieval, we construct a unified PubMed-derived corpus from approximately 20 million publicly available abstracts and 8 million full texts. Questions and corpus are encoded using Qwen3-Embedding-0.6B[4], and the corpus is indexed in Qdrant[5] for dense vector retrieval. Retrieved candidates are reranked under the original question using Qwen3-Reranker-8B[6]. A qualitative MeSH-colored visualization of the PubMed abstract embedding space, used only as a sanity check of the retrieval substrate, is provided in Supplementary Figure S2. The grounding threshold θ_g and confidence threshold θ_c used by the type-conditioned cascade controller are calibrated on a held-out development split and fixed at $\theta_g = 0.5$ and $\theta_c = 0.7$ for all reported experiments. Unless otherwise specified, all non-oracle baselines are implemented under the same retrieval corpus, embedding model, reranking model, answer backbone, decoding and formatting strategy, and evaluation pipeline. This controlled setup ensures that performance differences primarily reflect evidence assembly and control strategies rather than platform-level differences.

[2] https://vllm.ai
[3] https://huggingface.co/Qwen/Qwen3.5-35B-A3B
[4] https://huggingface.co/Qwen/Qwen3-Embedding-0.6B
[5] https://qdrant.tech
[6] https://huggingface.co/Qwen/Qwen3-Reranker-8B

Supplementary Note S6. Full algorithms and type-conditioned escalation

The grounded evidence sufficiency controller implements a single answer-form-conditioned escalation policy rather than a fixed global threshold or isolated question-specific rules. Each answer form is assigned an escalation configuration that specifies (i) which routing signal is most reliable for that answer space, (ii) whether a weak signal should trigger escalation, and (iii) whether the answer form should allow agentic assembly after the fast evidence path. Two signals are combined: the first-token log-probability of the draft, used as a lightweight confidence proxy, and an evidence-grounding check of the draft against the assembled evidence.

For *closed-form* answer types whose output is effectively a single token, such as yes/no answers and multiple-choice option letters, the first-token log-probability is a more reliable proxy for whole-answer confidence, and the policy therefore relies primarily on this signal. For *open-form* answer types whose output may contain multiple tokens or multiple entities, such as factoid entities, list answers, summaries, and expression sets, the first-token confidence is only a coarse screen because the model can be confident on the first token while later omitting entities, introducing unsupported aliases, or mismatching the required evidence substrate. For these answer forms, the grounding signal acts as the decisive safeguard, and a query is escalated when the draft is either low-confidence or insufficiently supported by the assembled evidence.

Some answer forms are assigned a non-escalating configuration when the development split indicates that forcing the agent route does not improve the answer and can perturb already-grounded fast-path predictions. Equivalently, answer forms are partitioned into a closed-form set $\mathcal{T}$closed, a non-escalating set $\mathcal{T}$no-esc, and the remaining open-form types. The decision in Algorithm S1 branches on this partition using the confidence threshold θ_c and grounding threshold θ_g. Both the partition and thresholds are fixed on the development split before final evaluation, rather than hand-set for individual question patterns. Algorithm S1 gives the full controller and Algorithm S2 gives the REPL-style iterative assembly invoked once the controller escalates.

Algorithm S1 Grounded Evidence Sufficiency Controller of BioHarness.

Require: question q, task type t, options O if applicable
Ensure: task-constrained answer a

$\mathcal{D} \leftarrow$ LiteratureEvidenceAssembly(q, t)
$\mathcal{G} \leftarrow$ ToolPrecall(q, t) # lightweight tool-derived grounding
$\mathcal{E}_{\text{fast}} \leftarrow$ MergeEvidence$(\mathcal{D}, \mathcal{G})$ # fast evidence context
$(a^{(0)}, \ell) \leftarrow$ DraftPrediction$(q, \mathcal{E}_{\text{fast}}; t, O)$
$c \leftarrow \exp(\ell)$ # confidence proxy
$e \leftarrow$ EvidenceGroundingCheck$(a^{(0)}, \mathcal{E}_{\text{fast}}, t)$ # evidence grounding signal
# type-conditioned escalation decision
if $t \in \mathcal{T}_{\text{no-esc}}$ **then**
  $\pi \leftarrow$ Fast # non-escalating answer form
else if $t \in \mathcal{T}_{\text{closed}}$ **then**
  $\pi \leftarrow$ Fast **if** $c \geq \theta_c$ **else** Agent # closed-form: confidence-driven routing
else
  $\pi \leftarrow$ Fast **if** $c \geq \theta_c \land e \geq \theta_g$ **else** Agent # open-form: confidence + groundedness
end if
if $\pi =$ Fast **then**
  return $a^{(0)}$ # fast evidence path
end if
$h \leftarrow$ REPLIterativeAssembly$(q, t, \mathcal{E}_{\text{fast}}, O; N_{\text{iter}})$ # Algorithm S2
$a \leftarrow$ FinalAnswer$(q,$ EvidencePack$(h, \mathcal{E}_{\text{fast}}); t, O)$
return a

Algorithm S2 REPL-style Iterative Evidence Assembly.

Require: question q, task type t, fast evidence context $\mathcal{E}_{\text{fast}}$, options O if applicable, iteration cap N_{iter}

Ensure: evidence trace h

Stage A – initialize tool and atlas evidence
$T \leftarrow \textsc{InitBiomedicalTools}(q, t)$ # callable grounding tools
$r \leftarrow \textsc{AtlasRouter}(q, t)$ # atlas query specification
if r.route_atlas **then**
    $A \leftarrow \textsc{FetchAtlasEvidence}(r.\text{query\_spec})$ # structured measurement evidence
else
    $A \leftarrow \varnothing$
end if
$h \leftarrow \textsc{InitContext}(q, t, \mathcal{E}_{\text{fast}}, T, A, O)$
# Stage B – iterative executable evidence assembly
for $i = 1, \ldots, N_{\text{iter}}$ **do**
    $\text{code}_i \leftarrow \textsc{CodeGeneration}(h)$ # ① code generation
    $\text{exec}_i \leftarrow \textsc{ReplExec}(\text{code}_i)$ # ② REPL execution
    $\text{obs}_i \leftarrow \textsc{ResultObservation}(\text{exec}_i)$ # ③ result observation
    $h \leftarrow h \| \text{code}_i \| \text{obs}_i$ # update evidence trace
    if $\textsc{IsFinal}(\text{obs}_i)$ **then**
        break # ④ stop if sufficient
    end if
end for
return h

Supplementary Note S7. Substrate overview and retrieval corpus

This note collects the conceptual and qualitative figures referenced from the main text. Figure S1 summarizes the three heterogeneous evidence substrates that motivate substrate-aware assembly, and Figure S2 provides a qualitative sanity check of the PubMed retrieval corpus used by the literature substrate.

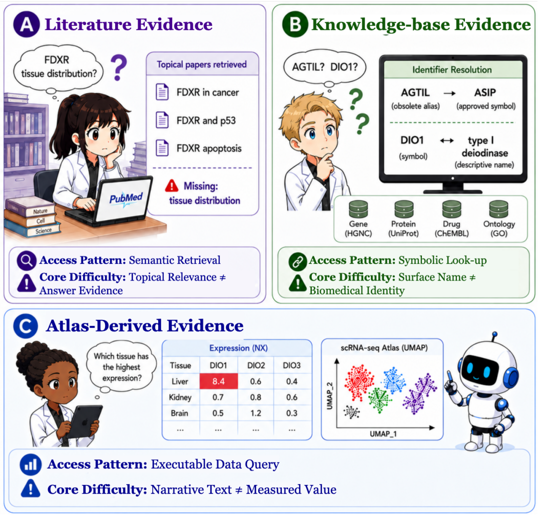

Figure S1: Heterogeneous evidence sources in biomedical question answering. Literature evidence supports claim-level reasoning through semantic retrieval, knowledge-base evidence supports entity-level grounding through identifier and relation lookup, and atlas-derived evidence supports measurement-level reasoning through executable queries over structured biological data.

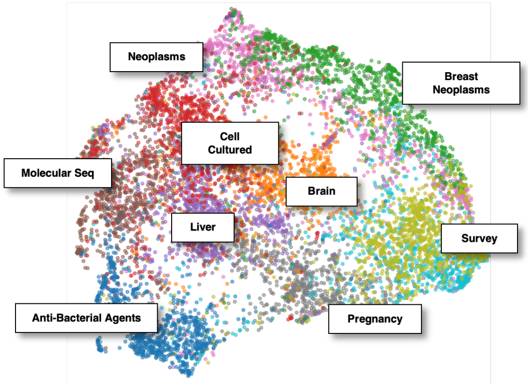

Figure S2: MeSH-colored subsampled visualization of the PubMed embedding space. Each point represents a corpus encoded by Qwen3-Embedding-0.6B, with colors assigned by article-associated MeSH terms and labels marking representative MeSH categories. This visualization is used only as a qualitative sanity check of the literature retrieval substrate.

Case I Dynamic Routing **PubMedQA (yes/no)**

$-C$ Ablation **Tie Performance / 7.4× Faster**

Q: *"Cholesterol screening in school children: is family history reliable to choose the ones to screen?"*

A: No

BioHarness's Routing Pipeline:

△ `Retrieved evidence (top-ranked literatures):`
[PMID 17971187]: "...Despite 64.4% of subjects reporting family history of hyperlipidemia, no relations between family history and serum lipid levels were found...."

△ `Evidence status:` answer explicitly supported by retrieved passage ✓

△ `Draft prediction:` **"No"** confidence > threshold

△ `Routing decision:` skip REPL execution (fast evidence path)

△ `Final answer:` "**No**" **Latency: 17.4 s**

BioHarness$_{-C}$: same final answer ("**No**"), but still executes a redundant multi-step agent loop before termination.

Overhead: 7.4× **Latency:** **129 s**

Figure S3: Cascade routing avoids unnecessary REPL-style iterative evidence repair.

Supplementary Note S8. Mechanistic case studies

This section provides the full annotated evidence traces for the three mechanistic cases. In the cascade-routing case, the PubMedQA evidence already supports a negative answer, so BioHarness terminates through the fast evidence path, whereas BioHarness$_{-C}$ forces a redundant agent trajectory. In the entity-grounding case, the deprecated alias `AGTIL` is not bridged by retrieved literature, while the Biomedical Tool Layer resolves it to the official gene symbol `ASIP`. In the measurement case, the retrieved abstracts for `FDXR` discuss disease mechanisms rather than tissue-level expression, while atlas-derived measurements provide the required structured substrate. The full traces are shown in Figures S3–S5.

Case II Biomedical Tool **GeneTuring (factoid)**

−T Ablation **Rescue Score 0 → 1**

Q: *"What is the official gene symbol of* ***`AGTIL`****?"*
(`AGTIL` is a deprecated alias of Agouti Signaling Protein.)
A: ASIP

BioHarness's Routing Pipeline:

△ `Retrieved evidence (top-ranked literatures):`

PMID	Title (excerpt)	AGTIL	ASIP
2045108	alanine: glyoxylate aminotrans.	×	×
9441744	serpin gene cluster chr 14q32	×	×
8954797	glycogen debranching (AGL) gene	×	×
23946868	GT198 in breast/ovarian cancer	×	×
18215257	RGST: rat gene symbol tracker	×	×
...			

△ `Evidence status:` no retrieved abstracts mention AGTIL

△ `draft prediction:` "unknown"

△ `Routing decision:` Escalate to Biomedical Tool Layer

△ `REPL trace with Biomedical Tools`

```
  >>> gene_resolver(''AGTIL'')
  >>> aliases: [AGTI, AGTIL,...]  # HGNC/MyGene alias lookup
  >>> official symbol: ASIP
```

△ `Final answer:` **"ASIP"** **Score = 1** **15.5 s**

BioHarness$_{-T}$:

△ `Retrieved evidence (top-ranked literatures):` ...

△ `draft prediction:` "unknown" confidence < threshold

△ `Routing decision:` Escalate to Further Literature Search

△ `REPL trace:`

search_pubmed("AGTIL gene")
lexical reformulations over 8 iterations
no alias bridge discovered in top-K retrieval

`Final answer:` **"Unknown"** **Score = 0** **17.9 s**

Figure S4: Biomedical Tool Layer resolves alias chains that literature retrieval cannot bridge.

Case III Atlas Evidence **SciHorizon-Gene (expression)**

−D Ablation **Rescue Score 0.00 → 80.0**

Q: *"What is the tissue-level expression pattern of the `FDXR` gene?"*

A: adrenal, testis, spleen, colon, ovary, skin, esophagus

BioHarness's Routing Pipeline:

△ `Retrieved evidence (top-ranked literatures):`

Retrieved Topic	PMID
FDXR DNA-damage signaling	12370809, 33113898
Oxidative-stress response	28842474
Mitochondrial apoptosis	11590433, 16528528
...	

△ `Evidence status:` The retrieved abstracts only cover FDXR disease mechanisms

△ `draft prediction:`
"colon and leukocytes" # confidence < threshold

△ `Router decision:`
Escalate to Atlas Evidence Layer

△ `REPL trace summary (details on the right panel):`
load HPA bulk atlas
→ read `FDXR.tissue_expression`
→ filter by `nx`≥1.0

△ `Final answer:` adrenal, testis, spleen, esophagus, ovary, skin, liver, brain **(6 / 7 GT recovered)**
Score = 80.0

Details of BioHarness's REPL Trace

```
>>> load_atlas("hpa_bulk")

>>> gene = "FDXR"
>>> rows = atlas['genes'][gene]
['tissue_expression']

>>> inspect(rows)
adrenal: 41.2; testis: 14.5; spleen: 6.8
esophagus: 4.1; ovary: 3.9; skin: 3.4;
liver: 2.2; brain: 1.6; colon: 0.7

>>> expressed = []

>>> for tissue, nx in rows:
>>>     if nx >= 1.0:
>>>       expressed.append(tissue)

>>> return_answer(expressed)
["adrenal", "testis", "spleen", "esophagus",
"ovary", "skin", "liver", "brain"] Elapsed: 2.3s
```

BioHarness$_{-D}$
The LLM generates the same draft answer from the retrieved evidence, but the BioHarness$_{-D}$ pipeline lacks an atlas substrate for structured per-tissue measurements.
`Final answer:` colon and leukocytes **F1 = 0.00**

Figure S5: Atlas-derived evidence provides structured tissue-level measurement grounding absent from the retrieved literature.

Supplementary Note S9. Prototype demo

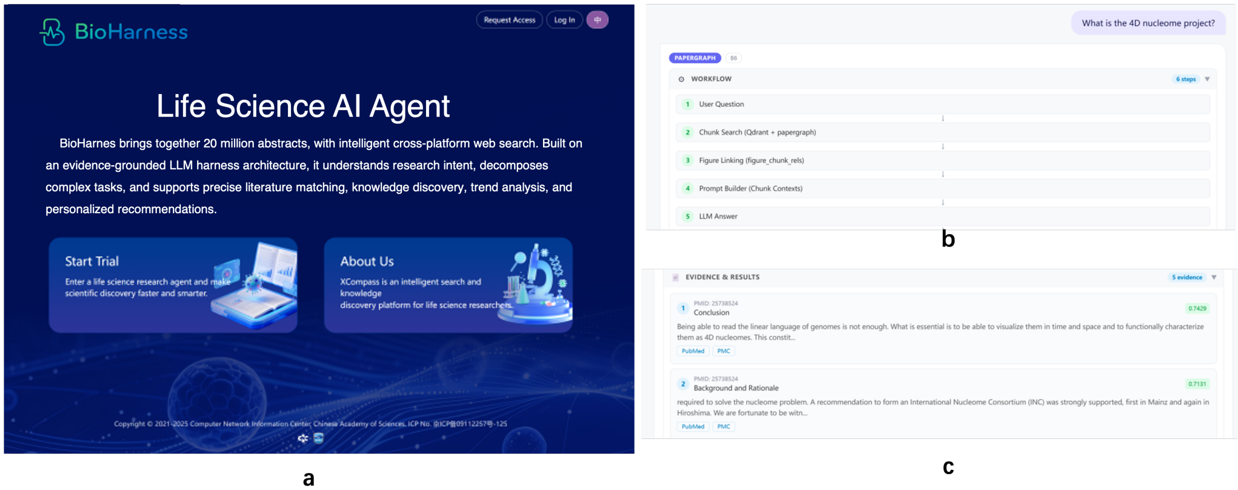

Figure S6: Prototype demo system of BioHarness. (a) The landing page provides access to the life-science question-answering interface. (b) The workflow panel visualizes the execution trace of a user query, including query intake, literature chunk search, figure or document linking, prompt construction, and answer generation. (c) The evidence panel displays retrieved literature snippets with document identifiers, source links, and relevance scores. The demo illustrates a practical instantiation of the harness and is not required for reproducing the benchmark results.

Supplementary Note S10. Frontier model and harness comparison

Models and settings. Standalone models (`claude-opus-5`, `gpt-5.6-sol`) were queried through OpenRouter with provider routing restricted to Anthropic and OpenAI, respectively. Harness runs used Claude Code 2.1.281 (with `claude-opus-5`) and Codex CLI v0.156.1 (with `gpt-5.6-sol`), each started in an empty working directory with a read-only sandbox and with all default settings. In the setting without web access, web search and fetch tools were disabled. BioHarness used the configuration of the main experiments (Qwen3.5-35B-A3B). In the web setting, the text of the pages fetched by Codex was passed to BioHarness's answer stage.
Sampling and scoring. We drew a stratified 5% sample of each of the nine benchmarks and used all 199 LitQA2 items. Final answers were extracted from harness outputs and evaluated using the same pipeline as BioHarness.
Benchmark leakage. With web access, Codex retrieved public copies of LitQA2 (e.g., the LAB-Bench dataset viewer) for 13 of the 199 items. These pages expose the labeled answer, and both Codex and the evidence-transplant arm, which receives the same pages, answered all 13 correctly. Excluding these items lowers the Codex score from 96.0 to 95.7 and the transplant score from 93.0 to 92.5. We identified benchmark copies by matching fetched URLs against known dataset hosts and searching the fetched text for verbatim question stems across all ten benchmarks.

References

Akari Asai, Zeqiu Wu, Yizhong Wang, Avirup Sil, and Hannaneh Hajishirzi. Self-rag: Learning to retrieve, generate, and critique through self-reflection. In *The Twelfth International Conference on Learning Representations*, 2023.

Boyu Chen, Zirui Guo, Zidan Yang, Yuluo Chen, Junze Chen, Zhenghao Liu, Chuan Shi, and Cheng Yang. Pathrag: Pruning graph-based retrieval augmented generation with relational paths. In *Proceedings of the AAAI Conference on Artificial Intelligence*, volume 40, pages 30183–30191, 2026.

Darren Edge, Ha Trinh, Newman Cheng, Joshua Bradley, Alex Chao, Apurva Mody, Steven Truitt, Dasha Metropolitansky, Robert Osazuwa Ness, and Jonathan Larson. From local to global: A graph rag approach to query-focused summarization. *arXiv preprint arXiv:2404.16130*, 2024.

Luyu Gao, Xueguang Ma, Jimmy Lin, and Jamie Callan. Precise zero-shot dense retrieval without relevance labels. In *Proceedings of the 61st Annual Meeting of the Association for Computational Linguistics (Volume 1: Long Papers)*, pages 1762–1777, 2023.

Zirui Guo, Lianghao Xia, Yanhua Yu, Tian Ao, and Chao Huang. Lightrag: Simple and fast retrieval-augmented generation. *arXiv preprint arXiv:2410.05779*, 2(3), 2024.

Xiaohan Huang, Meng Xiao, Chuan Qin, Qingqing Long, Jinmiao Chen, Yuanchun Zhou, and Hengshu Zhu. Scihorizon-gene: Benchmarking llm for life sciences inference from gene knowledge to functional understanding. In *Proceedings of the 32nd ACM SIGKDD Conference on Knowledge Discovery and Data Mining V. 2*, pages 9137–9148, 2026.

Zhengbao Jiang, Frank F Xu, Luyu Gao, Zhiqing Sun, Qian Liu, Jane Dwivedi-Yu, Yiming Yang, Jamie Callan, and Graham Neubig. Active retrieval augmented generation. In *Proceedings of the 2023 conference on empirical methods in natural language processing*, pages 7969–7992, 2023.

Di Jin, Eileen Pan, Nassim Oufattole, Wei-Hung Weng, Hanyi Fang, and Peter Szolovits. What disease does this patient have? a large-scale open domain question answering dataset from medical exams. *arXiv preprint arXiv:2009.13081*, 2020.

Qiao Jin, Bhuwan Dhingra, Zhengping Liu, William Cohen, and Xinghua Lu. PubMedQA: A dataset for biomedical research question answering. In Kentaro Inui, Jing Jiang, Vincent Ng, and Xiaojun Wan, editors, *Proceedings of the 2019 Conference on Empirical Methods in Natural Language Processing and the 9th International Joint Conference on Natural Language Processing (EMNLP-IJCNLP)*, pages 2567–2577, Hong Kong, China, November 2019. Association for Computational Linguistics. doi: 10.18653/v1/D19-1259. URL https://aclanthology.org/D19-1259/.

Qiao Jin, Yifan Yang, Qingyu Chen, and Zhiyong Lu. Genegpt: augmenting large language models with domain tools for improved access to biomedical information. *Bioinformatics*, 40(2):btae075, 2024.

Vladimir Karpukhin, Barlas Oguz, Sewon Min, Patrick Lewis, Ledell Wu, Sergey Edunov, Danqi Chen, and Wen-tau Yih. Dense passage retrieval for open-domain question answering. In *Proceedings of the 2020 conference on empirical methods in natural language processing (EMNLP)*, pages 6769–6781, 2020.

Anastasia Krithara, Anastasios Nentidis, Konstantinos Bougiatiotis, and Georgios Paliouras. Bioasq-qa: A manually curated corpus for biomedical question answering. *Scientific Data*, 10(1): 170, 2023.

Jon M Laurent, Joseph D Janizek, Michael Ruzo, Michaela M Hinks, Michael J Hammerling, Siddharth Narayanan, Manvitha Ponnapati, Andrew D White, and Samuel G Rodriques. Lab-bench: Measuring capabilities of language models for biology research. *arXiv preprint arXiv:2407.10362*, 2024.

Mingchen Li, Halil Kilicoglu, Hua Xu, and Rui Zhang. Biomedrag: A retrieval augmented large language model for biomedicine. *Journal of Biomedical Informatics*, 162:104769, 2025.

Chin-Yew Lin. Rouge: A package for automatic evaluation of summaries. In *Text summarization branches out*, pages 74–81, 2004.

Ankit Pal, Logesh Kumar Umapathi, and Malaikannan Sankarasubbu. Medmcqa: A large-scale multi-subject multi-choice dataset for medical domain question answering. In *Conference on health, inference, and learning*, pages 248–260. PMLR, 2022.

Chuan Qin, Xin Chen, Chengrui Wang, Pengmin Wu, Xi Chen, Yihang Cheng, Jingyi Zhao, Meng Xiao, Xiangchao Dong, Qingqing Long, et al. Scihorizon: Benchmarking ai-for-science readiness from scientific data to large language models. In *Proceedings of the 31st ACM SIGKDD Conference on Knowledge Discovery and Data Mining V. 2*, pages 5754–5765, 2025.

Pranav Rajpurkar, Jian Zhang, Konstantin Lopyrev, and Percy Liang. Squad: 100,000+ questions for machine comprehension of text. In *Proceedings of the 2016 conference on empirical methods in natural language processing*, pages 2383–2392, 2016.

Xinyi Shang, Xu Liao, Zhicheng Ji, and Wenpin Hou. Benchmarking large language models for genomic knowledge with geneturing. *Briefings in Bioinformatics*, 26(5):bbaf492, 2025.

Harsh Trivedi, Niranjan Balasubramanian, Tushar Khot, and Ashish Sabharwal. Interleaving retrieval with chain-of-thought reasoning for knowledge-intensive multi-step questions. In *Proceedings of the 61st annual meeting of the association for computational linguistics (volume 1: long papers)*, pages 10014–10037, 2023.

Chengrui Wang, Qingqing Long, Meng Xiao, Xunxin Cai, Chengjun Wu, Zhen Meng, Xuezhi Wang, and Yuanchun Zhou. Biorag: A rag-llm framework for biological question reasoning. *arXiv preprint arXiv:2408.01107*, 2024.

Wenhao Yu, Hongming Zhang, Xiaoman Pan, Peixin Cao, Kaixin Ma, Jian Li, Hongwei Wang, and Dong Yu. Chain-of-note: Enhancing robustness in retrieval-augmented language models. In *Proceedings of the 2024 conference on empirical methods in natural language processing*, pages 14672–14685, 2024.

Yuxin Zuo, Shang Qu, Yifei Li, Zhangren Chen, Xuekai Zhu, Ermo Hua, Kaiyan Zhang, Ning Ding, and Bowen Zhou. Medxpertqa: Benchmarking expert-level medical reasoning and understanding. *arXiv preprint arXiv:2501.18362*, 2025.
